# Histologic effects of demineralized bone matrix on regeneration of alveolar socket in diabetic rats

**Published:** 2009-01-07

**Authors:** Mahdi Kadkhodazadeh, Fatemeh Mollaverdi, Hamid Reza Abdolsamadi, Ramin Azar, Madjid Ghasemian Pour, Shahriyar Ahmadpour

**Affiliations:** 1 *Department of Periodontics, Dental School, Hamadan University of Medical Sciences, Hamadan, Iran.*; 2 *Dentist, Hamadan, Iran.*; 3 *Department of Oral Medicine, Dental School, Hamadan University of Medical Sciences, Hamadan, Iran.*; 4 *Department of Orthodontic, Dental School, Hamadan University of Medical Sciences, Hamadan, Iran.*; 5 *Orthodontist, Dental Research Center, Iranian Center for Endodontic Research, Shahid Beheshti University of Medical Sciences, Tehran, Iran.*; 6 *Histology and Embryology Department, Mashad University of Medical Sciences, Mashad, Iran.*

**Keywords:** Diabetes, Demineralized bone matrix, Microscopy, Tooth socket

## Abstract

**INTRODUCTION:** The aim of this *in vivo* study was to determine the effect of demineralized bone matrix (DBM) on alveolar bone repair in type I diabetic rats.

**MATERIALS AND METHODS:** This study was carried out on 40 adult (8 weeks-old) albino rats with an average weight of 200-250 grams. The animals were divided into four groups (n=10) as follows: group 1 nondiabetic rats, group 2, 3 and 4 were diabetic rats, group 4 rats took one unit of insulin daily. Diabetes was induced by Alloxan Monohydrate through the tail veins of the rats in groups 2-4. Only group 4 received insulin NPH 1 unit daily. After 10 days, the upper right incisors of all samples were extracted and the socket was filled with DBM in groups 3 and 4. The animals were sacrificed at the end of week 1 and 2. The specimens were prepared and stained with H&E.

**RESULTS:** Histological results of group 4 displayed osteoblastic activity and bone formation with collagen fibers at the end of the first week and thick bone trabeculae formation in vicinity of DBM at the end of second week. In group 3, DBM showed some osteoinductivity at the end of the first week, but in some regions DBM particles were degraded by osteoclastic activity. Bone trabeculae formed with a dispersed and separate pattern at the end of second week. In group 2 hematoma and inflammation were dominant histological features at the end of first and second weeks; poor bone formation was detected in these two groups (2 and 3). In group 1, the results were as expected.

**CONCLUSION:** It seems demineralized bone matrix simulate osteoblastic activity.

## INTRODUCTION

The two major types of diabetes are *type 1, *formerly known as "insulin-dependent diabetes mellitus" (IDDM) and *type 2 *formerly called "non-insulin-dependent diabetes mellitus (NIDDM)". During the past decade, medical management of diabetes has changed significantly to minimize the debilitating complications associated with this disease. Type 1 diabetes is one of the metabolic diseases that involve increase in blood sugar and disturbance in carbohydrate, lipid and protein metabolism (1). Decrease in bone repair and formation are one of the consequences and signs of diabetes (2). Demineralized freeze dried bone allograft (DFDBA) or demineralized bone matrix (DBM) and autogenous grafts are some of the used materials in guided bone regeneration (GBR) (3-5). DBM is one of the allograft materials employed in periodontal surgery, bone regeneration, infection and trauma (6), first utilized by Urist (7).

**Figure 1:**
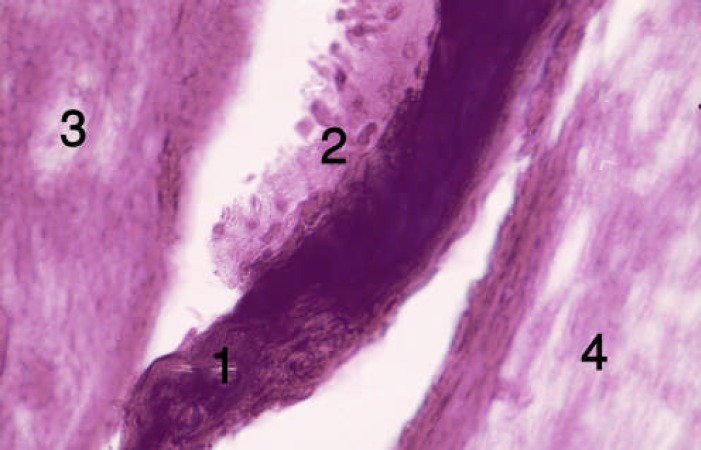
Tissue sections from the alveolar socket of the forth group [first week]. 1) DBM, 2) osteoblasts, 3) Forming bone, 4) Collagen

This material had great success in repair of calvarial bone defects (8). There have been controversial reports regarding the induction and guided bone regeneration ability of DBM materials; however the increased popularity of synthetic materials has limited the use of DBM in maxillofacial surgeries (3). Induction and guided bone formation of DBM may be due to the presence of bone morphogenic protein (BMP). These proteins are considered important factors in the formation of limbs in addition to their key role in bone regeneration (9). Investigations to introduce and recognize DBM as a grafts material are still in process (10). Hence the aim of this in vivo study was to determine the effects of DBM on alveolar bone repair of diabetic rats (type I).

## MATERIALS AND METHODS

This *in vivo* study was performed according to other similar studies (8,11-13). All experiments were conducted according to the guideline of local animal use and care committees and executed according to national animal law.

Forty 8-week old albino rats with an average weight of 200-250 grams were selected and randomly divided into 4 groups of 10 each (11). Group 1 was non diabetic and the other 3 groups were diabetic. For creating diabetes in diabetic groups, we diluted a vial of monohydrate Alexon (St. Louis, MO, USA) with buffer saline, then injected 52 mg/kg of this solution to the rats immediately after its preparation through tail veins by insulin syringe (12).

Five days after injection, blood samples were derived through retro-orbital venous sinus by means of micropipette in samples of groups 2, 3, and 4. Blood sugar level showed an increase from 7 mmol (normal) before injection to 13 mmol after injection. During treatment, blood sugar was kept stable at approximately 7 mmol. Rats in group 4 (insulin treated, diabetic) took 1 unit of NPH insulin daily (11).

**Figure 2:**
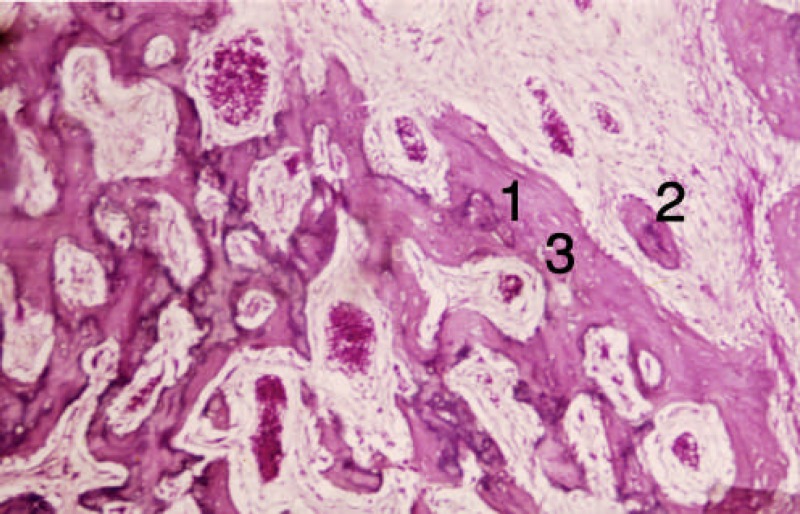
Section from the trabecular bone of the forth group [second week]. 1) Formed trabeculas, 2) Osteoblasts, 3) Osteocytes

Ten days after diabetes induction, general anesthesia was performed by 10mg/kg Ketamin HCl (Alfanso, the Netherland) and 1mg/kg chlorpromazine injection, and then upper right incisor of each rat was extracted. After hemostasis was achieved in groups 3 and 4, the dental sockets were filled with mixed saline and DBM, the area was then sutured. In groups 1 and 2 dental sockets were not filled. Finally, 2 mL of pentabioticveternario (sigma type antibiotic) was injected intramuscularly (11).

At the end of two weeks, five rats from each group was randomly separated and beheaded under general anesthesia and placed in 10% formalin. After that, decalcification was performed by means of formic and chloridric acids (Kiyankaveh, Iran) for one week and 5-µm tissue sections were prepared and stained by hematoxylin and eosin (Padtan Teb Inc., Tehran, Iran).

In this study, Kim’s method was used to harvest DBM (13). After separating long femoral and tibia bones of 20 rats, the end of these bones were placed in cold sterile distilled water, the bone marrow was extruded and the attached tissues from bone surfaces were separated. The following steps were performed sequentially:

1) Immersion of the specimens in absolute pure ethanol for 1 hour; 

2) Immersion in ethyl ether for half an hour;

3) Placing it in an oven (temperature of 36^º^C)

**Figure 3:**
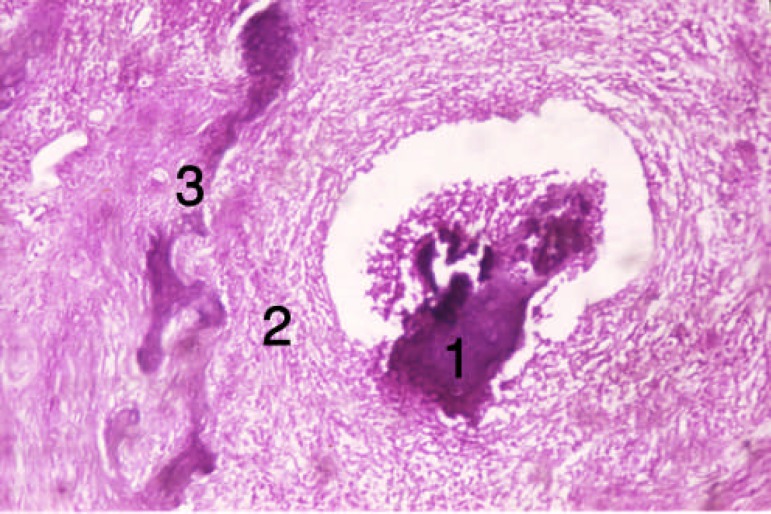
Tissue section from the alveolar socket of the third group [first week]. 1) DBM, 2) Cellular infiltration, 3) Forming bone trabeculas

**Figure 4:**
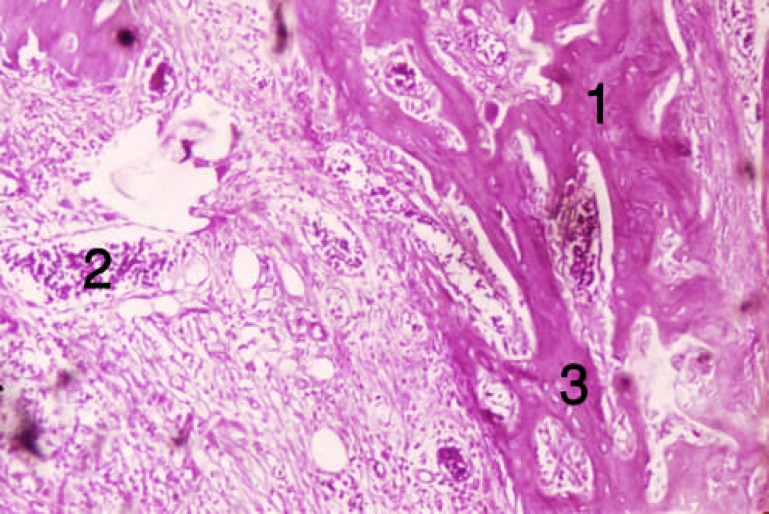
Tissue section from trabecular bone of the third group [second week]. 1) Bone trabeculas, 2) Vessels, 3) Osteocytes

**Figure 5:**
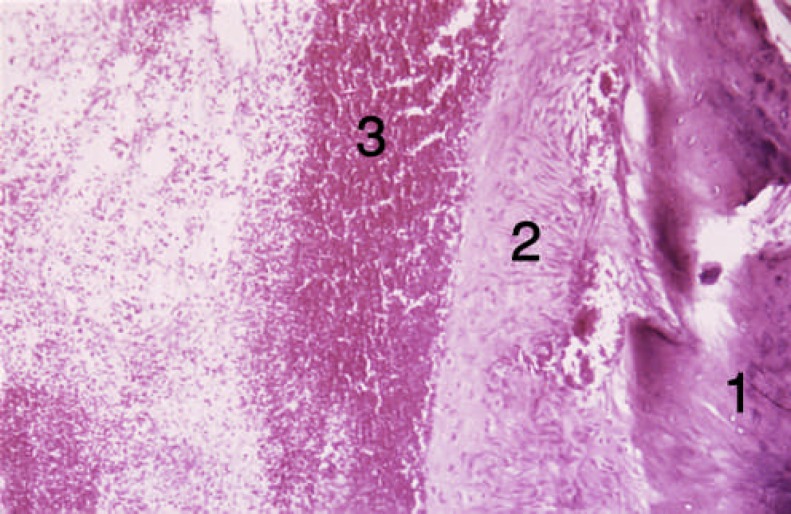
Tissue section from the alveolar socket of the second group [First week]. 1) Bone, 2) Connective tissue, 3) Hematoma

**Figure 6:**
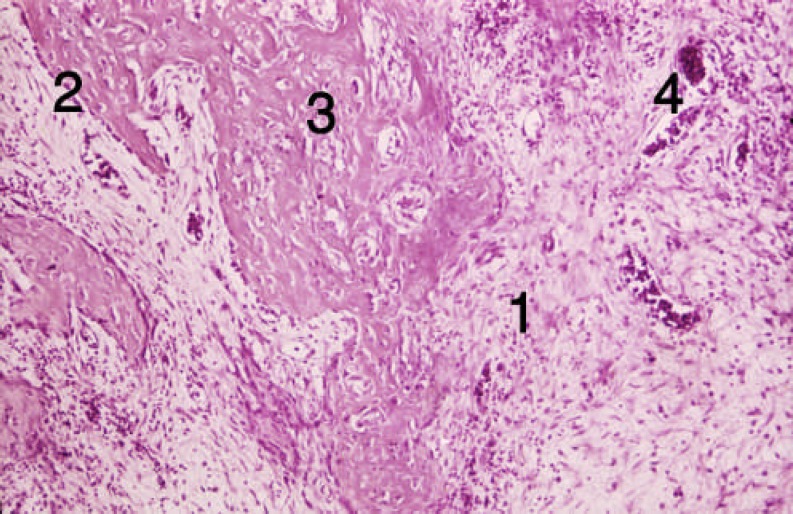
Tissue section from the formed bone in the alveolar socket of the second group [second week]. 1) Connective tissue, 2) Osteoblasts, 3) Osteocytes, 4) Vessels

for 1 night;

4) Milling and powdering the bone;

5) Immersing in 0.5% normal chloridric acid solutions for 3 h and then irrigating with water;

6) Immersing in ethanol solution for 1 hour and in ether for half an hour;

7) Putting in oven in the temperature of 36^º^C for 1 night;

8) Keeping the prepared material in the temperature of 2-4^º^C.

All mentioned procedures were performed in the sterile condition.

## RESULTS

At the end of the first week, osteoblastic activity and osseous trabeculas formation occurred around DBM and collagen fibers in group 4 (insulin treated). DBM particles were detectable with sharp borders and acellular surface or empty lacuna. These changes were clearly evident in four animals (80%). At the end of the second week, intramembranous bone, vessel and connective tissue formation occurred (Figure 1and Figure 2).

In the third group (diabetic and DBM), inflammation and cellular infiltration occurred at the end of the first week. Limited bone formation around DBM particles was detected; activated and DBM fragmenting macrophages and osteoclasts were also observed (two rats, 40%). However, no changes occurred in some areas. In this group, new-formed trabecula, connective tissue and lots of dilated blood vessels were seen at the end of the second week (Figure 3 and Figure 4). In group 2 (diabetic), hematoma was the dominant tissue profile at the end of the first week. At the end of the second week, limited bone formation, connective tissue, dilated vessels and intramembranous bone formation occurred (Figure 5 and Figure 6).

**Figure 7:**
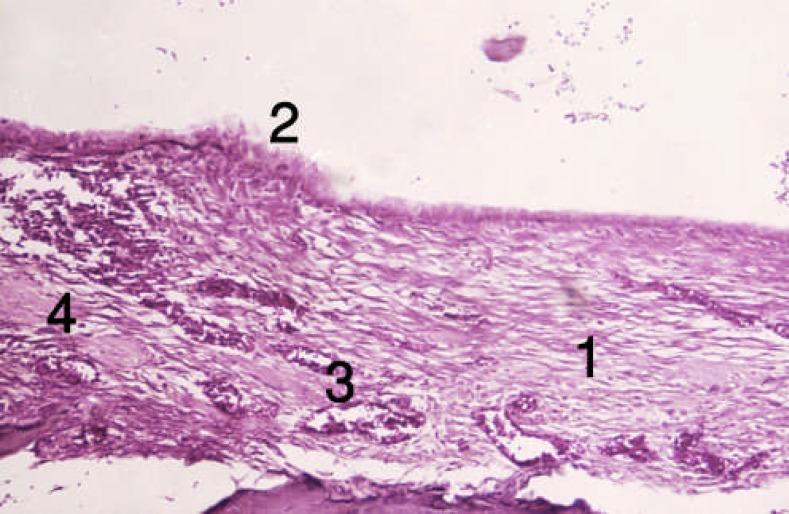
Tissue section from the alveolar socket of the control group [First week]. 1) Connective tissue, 2) Cells during conversion to osteoblast, 3) Vessels, 4) Fine forming trabeculas

**Figure 8:**
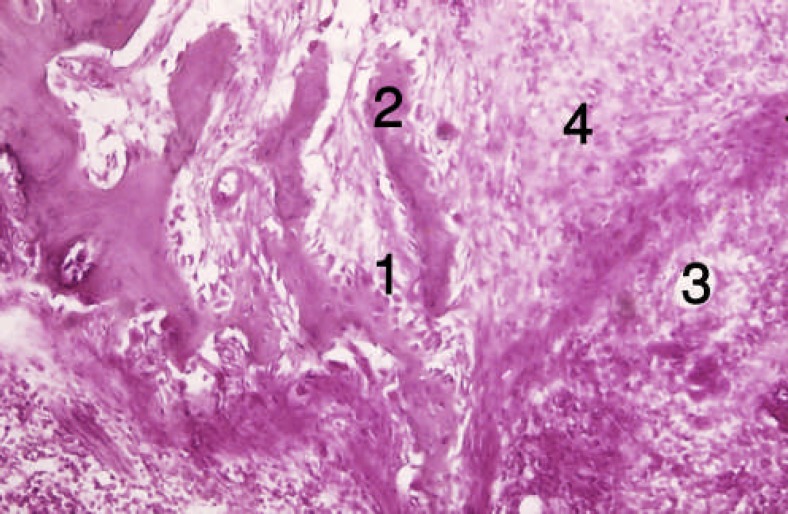
Tissue section from the alveolar sockets osseous trabecula of the control group [second week]. 1) Osteoblast, 2) Formated bone, 3) Vessels, 4) Connective tissue

In group 1, bone formation was limited to periodontal ligament tissue area around the extracted tooth at the end of the first week; in flammation and dilated vessels were also observed. At the end of second week, bony islands formed by means of intramembranous bone formation, dilated vessels, new osteoblasts; connective tissue could also be seen (Figures 7, 8)

## DISCUSSION

In diabetic rats treated with insulin (group 4), DBM had favorable effects on GBR. This caused the DBM particles to be surrounded by osteoblast cells and osteoid. Group 3 showed more bone formation than group 2; therefore we can conclude that DBM may induce effective GBR. According to these results DBM had a little inductive effect on the untreated diabetic group (group 4); this may be due to other factors such as pH changes and protein catabolism which will decrease its effects*.*

It also seems that the control of blood sugar with insulin is an effective measure to decrease the severity of inflammation and protein catabolism in group 4 and increase the bone tissue formation. Final glycolysis products will have a negative effect on bone repair and it decreases bone formation. Chay *et al.* showed that mesenchymal cells, preosteoblasts and osteocytes could be seen around DBM particles on the 15^th^ day when implanting DMB in the cranium (14). In the control group, some changes were observed in PDL cells though no osteoblast and osteoid material was detected. It seems that DBM particles may induce bone formation by attracting multipotential cells and stimulating their differentiation to osteoblasts.

Some reports claim that bone formation around these particles is due to cytokine release from DBM and invasion of multipotential cells to the injured area (15). It is likely that DBM releases cytokines or DMPs and that the cavities present in the DBM particles take part in biomineralization. Note that this mechanism requires important factors such as collagen, appropriate pH and hydroxyl groups that become defective in diabetes (2,16). Even though DBM has good ability to form bone and is an appropriate graft material; more investigations are still required (10).

At the end of the first week, fine trabeculae were seen around DBM particles in group 3; cellular infiltration and inflammation were also dominant. At the end of the second week, minor osteogenesis became visible in some areas. Due to the inflammatory activity, destruction and fragmentation of DBM particles, limited ontogenesis also occurred. In this group at the end of the second week, cellular infiltration was still present around bone, although osteogenesis activity was detected in non-treated areas. DBM effects on guided osteogenesis were seen in 40% of our samples; even though in some reports, unpredictable biologic behavior of DBM is mentioned (16). However, DBM osteogenesis ability in alveolar socket has been focused by Callan (3), but DBM effectiveness has been decreased to some extent because of metabolic changes and when inflammation superimposes. Samples in this group had obviously delayed osteogenesis.

In group 1, at the end of the first week, osteogenesis was observed with peripheral to

central direction in some parts of remained PDL. Current up to date research has demonstrated that rapid osteogenesis after tooth extraction in normal tissue is the result of PDL collagen destruction as well as the fibronectin effect.

## CONCLUSION

This study showed that DBM can have inductive effects in rats with controlled diabetes; DBM seems to stimulate osteoprogenitor cells to differentiate into osteoblasts. A good recommendation would be to carry our further studies with DBM in diseased conditions and control the qualitive and quantitive factors.
